# Determining a Relationship Between Applied Occlusal Load and Articulating Paper Mark Area

**DOI:** 10.2174/1874210600701010001

**Published:** 2007-07-23

**Authors:** Jason P Carey, Mark Craig, Robert B Kerstein, John Radke

**Affiliations:** a Ph.D., 4-9 Mechanical Engineering Building, University of Alberta, Edmonton, Alberta, T6G 2G8, Canada; b B.Sc., Department of Mechanical Engineering. University of Alberta, Edmonton, Alberta, T6G 2G8, Canada; c DMD and Certificate in Prosthodontics; Former assistant clinical professor, Department of Restorative Dentistry, Tufts University School of Dental Medicine; d 4113 North Port Washington Road Milwaukee, WI, USA

**Keywords:** Mark Area, Applied Occlusal Load, Articulating Paper Appearance, MTS Uniaxial Test Machine

## Abstract

Articulating paper mark size has been widely accepted in the dental community to be descriptive of occlusal load. The objective of this study is to determine if any direct relationship exists between articulating paper mark area and applied occlusal load. A uniaxial testing machine repeatedly applied a compressive load, beginning at 25N and incrementally continuing up to 450N, to a pair of epoxy dental casts with articulating paper interposed. The resultant paper markings (*n* = 600) were photographed, and analyzed the mark area using a photographic image analysis and sketching program. A two-tailed Student’s *t-*test for unequal variances compared the measured size of the mark area between twelve different teeth (p < 0.05). Graphical interpretation of the data indicated that the mark area increased non-linearly with increasing load. When the data was grouped to compare consistency of the mark area between teeth, a high variability of mark area was observed between different teeth at the same applied load. The Student’s t-test found significant differences in the size of the mark area approximately 80% of the time. No direct relationship between paper mark area and applied load could be found, although the trend showed increasing mark area with elevating load. When selecting teeth to adjust, an operator should not assume the size of paper markings, accurately describing the markings’ occlusal contact force content.

## INTRODUCTION

Articulating paper is commonly used by the dental community to identify contact points between the maxillary and mandibular teeth during all forms of natural tooth occlusal adjustments and dental prosthesis insertions. These corrective adjustments are made by selectively grinding the paper marks to obtain occlusal stability [1], multiple contacts throughout the arches that exhibit simultaneity [2], and reduced stress on the occlusal contacts and the periodontium [3]. The selected marks to adjust are generally chosen based on their appearance characteristics.

During occlusal adjustment procedures, to aid articulating paper marks in the determination of which teeth and con-tact(s) require adjustment, clinical use of shimstock foil (Almore International; Portland, OR, USA) has been advocated in combination with articulating paper markings [4]. The strips are “tugged” between occluded teeth to subjectively determine the strongest “holding” contacts, after which articulating paper is used to mark the isolated teeth for adjustment. It has been observed that shim stock removal forces in small occlusal spacing gaps showed no significant difference [4]. Contact “hold” resistance levels are subjective. Therefore, it is a difficult guiding factor to utilize, when selecting contacts to adjust the demonstrated variable forces within occlusal contacts. And, because shim stock foil does not mark the selected teeth, the articulating paper markings are the primary guide for the operator when selecting which contact(s) require adjustment.

It has been advocated in textbooks on Occlusion [2,3,5,6,7] that mark area is a representative of the load contained within the mark. Legends to photographs depicting occlusal adjustment technique end results and paper mark appearance describe that large and dark marks indicate heavy load, and that smaller and light marks indicate lesser loads [5,6,7]. Additionally, the presence of many similar sized marks spread around the contacting arches is purported to indicate equal occlusal contact intensity, evenness, and simultaneity [1,3].

However, a recent publication describing the utilization of paper markings with a computerized occlusal analysis system (T-Scan II for Windows; Tekscan, Inc, Boston, Mass. USA) to establish clinically measurable bilateral simultaneous contacts, illustrated that, multiple similarly sized paper marks, spread around the arch, did not actually demonstrate measurable contact simultaneity [8]. This same computer system has been shown to reproduce applied load for up to 20 users [9] while accurately measuring the individual force content of the contacts represented by articulating paper markings [9]. The paper markings in this force reproduction analysis that were attempting to illustrate simultaneity, when analyzed by the computer’s graphical display of relative force measurements of the individual tooth contacts, showed that variable load was observed in those same, similar sized marks that could not accurately represent contact simultaneity [9]. In light of this information, it is quite possible that the relative size of differing articulating paper mark areas may not accurately predict occlusal contact force characteristics.

Published studies about articulating paper [10,11] are analyses of the physical properties of the papers themselves (thickness, composition, ink substrate, plastic deformation), and offer no evidence to suggest that variable articulating paper mark areas can describe variable occlusal loads. The published concepts that relate paper mark area to its load content are predicated upon the idea that the size of the markings indicate the range of the applied occlusal loads.

The proposed experimental design of this bench analysis attempted to isolate *articulating paper Mark Area* as the sole variable measured during an occlusal contact marking procedure. The purpose of this study is to determine if a relationship exists between the occlusal load applied to non-wearing epoxy casts and the size of the markings produced from tooth contact when a clinically used dental articulating paper is interposed.

## MATERIAL AND METHODS

To evaluate effect of occlusal loads on articulating paper mark area solid epoxy casts (model #s MJ567 and MJ568, Columbia Dentoform, Long Island, NY, USA) with no soft tissue components, were employed.

**Fig. (1):**
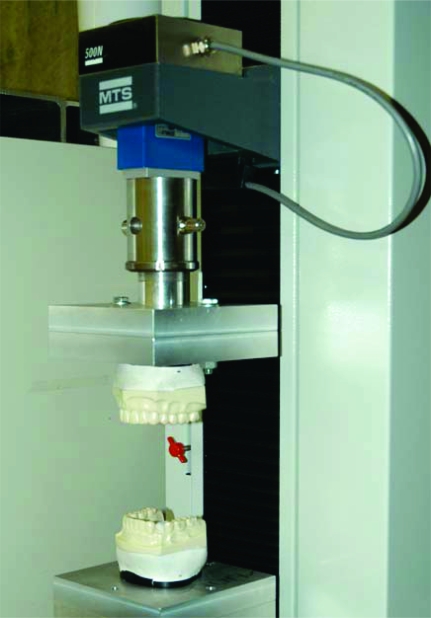
MTS testing machine- open prior to test.

**Fig. (2):**
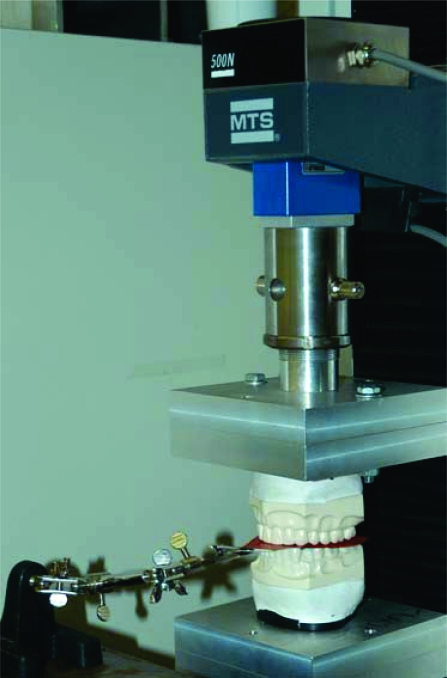
MTS testing machine - closed with casts intercuspated and articulating paper horseshoe interposed.

Vertical loading was accomplished by designing a cast anchoring apparatus that attached the epoxy casts to a MTS uniaxial testing machine (MTS Systems Corporation, Eden Prairie, MN, USA.) (Figs. **1** and **2**). The dental cast base plates were secured to the MTS by means of machined rods with alignment holes that ensured precise alignment of the maxillary and mandibular casts prior to testing. Once secured to the base plates, the casts were rigidly anchored during all cast intercuspation testing.

The MTS has a self-calibrating/zeroing program that was calibrated and zeroed prior to data collection. The crosshead, which is the part of the MTS that travels up and down to intercuspate the casts, was initially positioned to leave sufficient space for the thickness of the articulating paper (Horseshoe/Full Arch, red/blue articulating film; Ardent, Inc., Os-sining, NY, USA). Preliminary loading of the casts was performed once to properly mate the casts and to secondly ensure that the overshoot of the load cell was an acceptable value. A single 63 micron (0.0025 in.) thick horseshoe was then inserted between the casts covering both arch occlusal surfaces, red surface up/blue surface down, while being held in place by the clamps (Fig. **1**). A 25-mm/min strain rate was used for all tests. The loading began before the casts were intercuspated until complete intercuspation at varying and specified loads. The displacement of the crosshead was quickly recorded and the operator returning the crosshead automatically to the zero position released the load. The displacement of the load cell and selected applied load per cast “*tap*” were recorded from the *Results Window* in the MTS control program. This procedure was repeated twice more to simulate the tapping of the teeth together 3 times, as is done the intraoral marking procedure. Each 3-tap trial comprised one test.

**Fig. (3):**
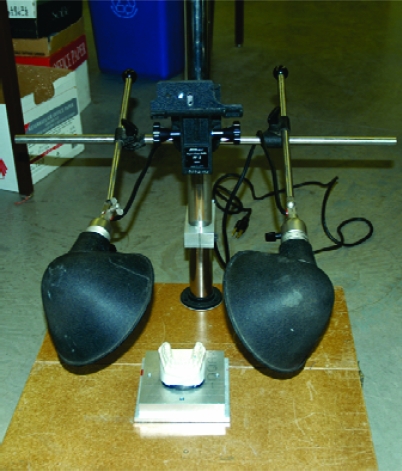
Photographic set up–camera placed at 90° directly over cast.

Photographs of the paper markings left on the maxillary and mandibular casts resultant from each 3-tap trial were obtained post-test by removing the 2 casts from the MTS and affixing them individually to a photographic alignment jig. A camera mount and locating plate system, manufactured by the Department of Mechanical Engineering at the University of Alberta, precisely secured a camera in the same position for every photograph. A tripod (model #200 Canon Deluxe, Cannon Inc., Lake Success, NY, USA) was modified to prevent motion by being affixed to an aluminum locating plate. The locating plate was made from a 1″ (25.4mm) thick aluminum, with alignment dowel pins and three clamps to position the dental cast base plates. This produced dimensionally and perspectively consistent photos (Fig. **3**). A 6-mega pixel digital camera was used (Nikon D100, Nikon Corp, Melville, NY, USA.) Focus and magnification were consistent for every test. The shutter speed (1/80 sec or 0.0125 seconds) and aperture (f/8) of the camera were kept constant. No camera flash was employed to reduce the variability in the timing of the flash. Instead, lamps provided constant and consistent lighting.

After the marked casts were affixed to the locating plate (Fig. **3**) one picture per cast was taken. The markings on the casts were then removed using rubbing alcohol, paper towel, and a toothbrush to avoid all cross contamination in the following test. Casts were given ample time to dry before the next test.

Once dry, the casts were replaced into the MTS and at the same load and retested. Each 3–tap trial was photographed before the next 3-tap trial. Five 3–tap trials per load were tested and photographed. The load was then increased 25 - 50N, and the entire was process repeated. Within a simulated human occlusal force range from 0 N to 522 N [12, 13], the casts were loaded at 25, 50, 100, 150, 200, 250, 300, 350, 400, and 450 N. The experimental design produced 100 photos for analysis. In all photographs, 6 consistent markings (indicating 6 contacts) were identified on both casts. Any other inconsistent occlusal markings were disregarded. This was a subjective assessment of the remaining markings that was designed to exclude indiscriminate marks that were not clearly repetitive occlusal contacts.

**Fig. (4):**
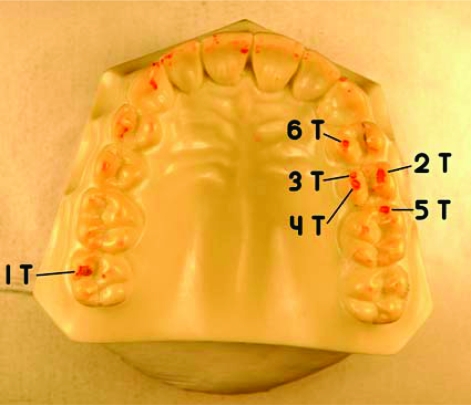
Maxillary cast – 6 consistent red articulating paper markings.

**Fig. (5):**
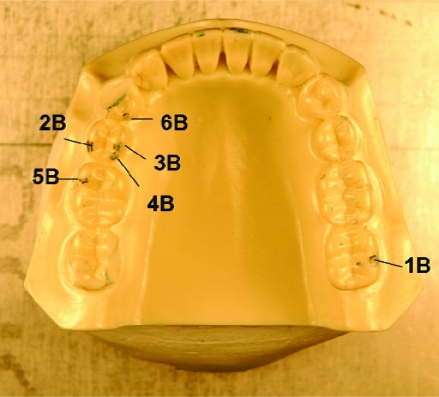
Mandibular cast–6 consistent blue articulating paper markings.

The 12 distinct contact markings (Figs.**4** and **5**) were analyzed using *ImageJ* software (developed at the National Institutes of Health, Washington, DC, USA) to magnify the markings so that the *ImageJ Freehand Sketcher* could be used to trace the boundary of the markings. The *ImageJ Measure Command* assessed the number of pixels enclosed within the area of the sketch. The markings were analyzed sequentially; from contact numbers 1 - 6. A total of 600 (*n* = 12 teeth x 10 force levels x 5 repetitions = 600) marks were statistically analyzed.

## RESULTS

**Fig. (6):**
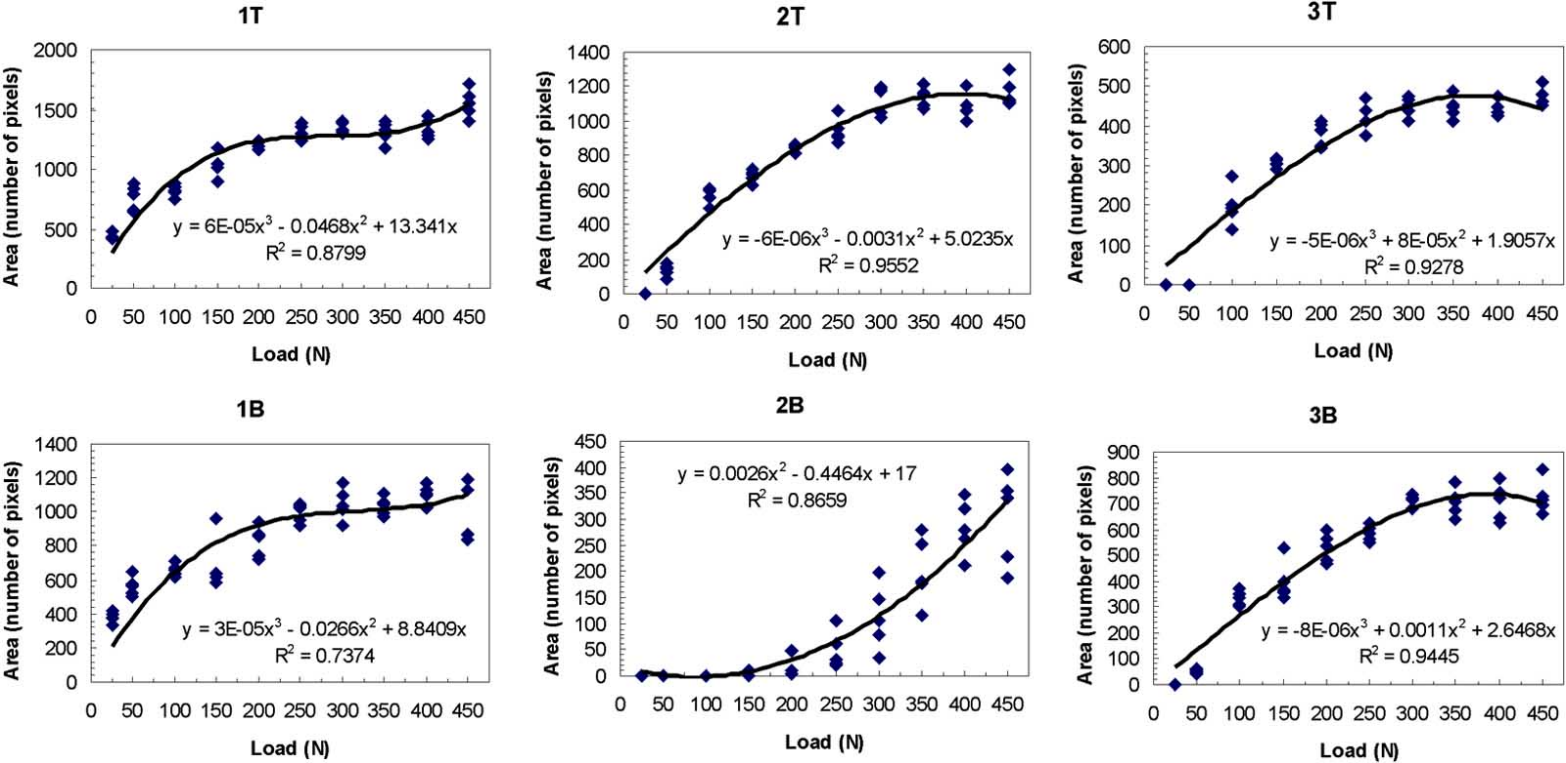
Mark area *vs*. specified maximum load curves – upper (T) and lower (B) contacts #s 1–3.

**Fig. (7):**
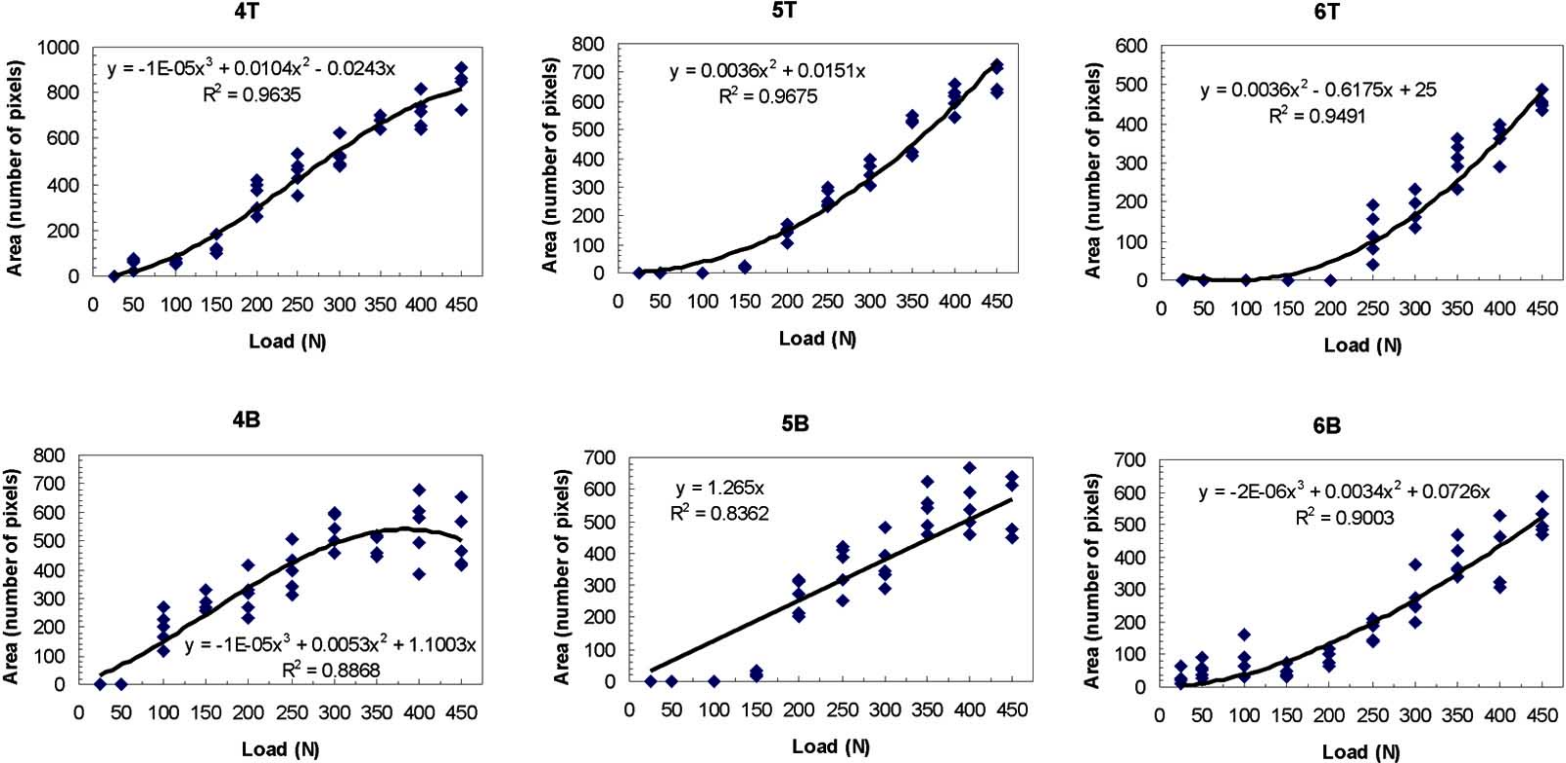
Mark area *vs*. specified maximum load curves–upper (T) and lower (B) contacts #s 4–6.

**Fig. (8):**
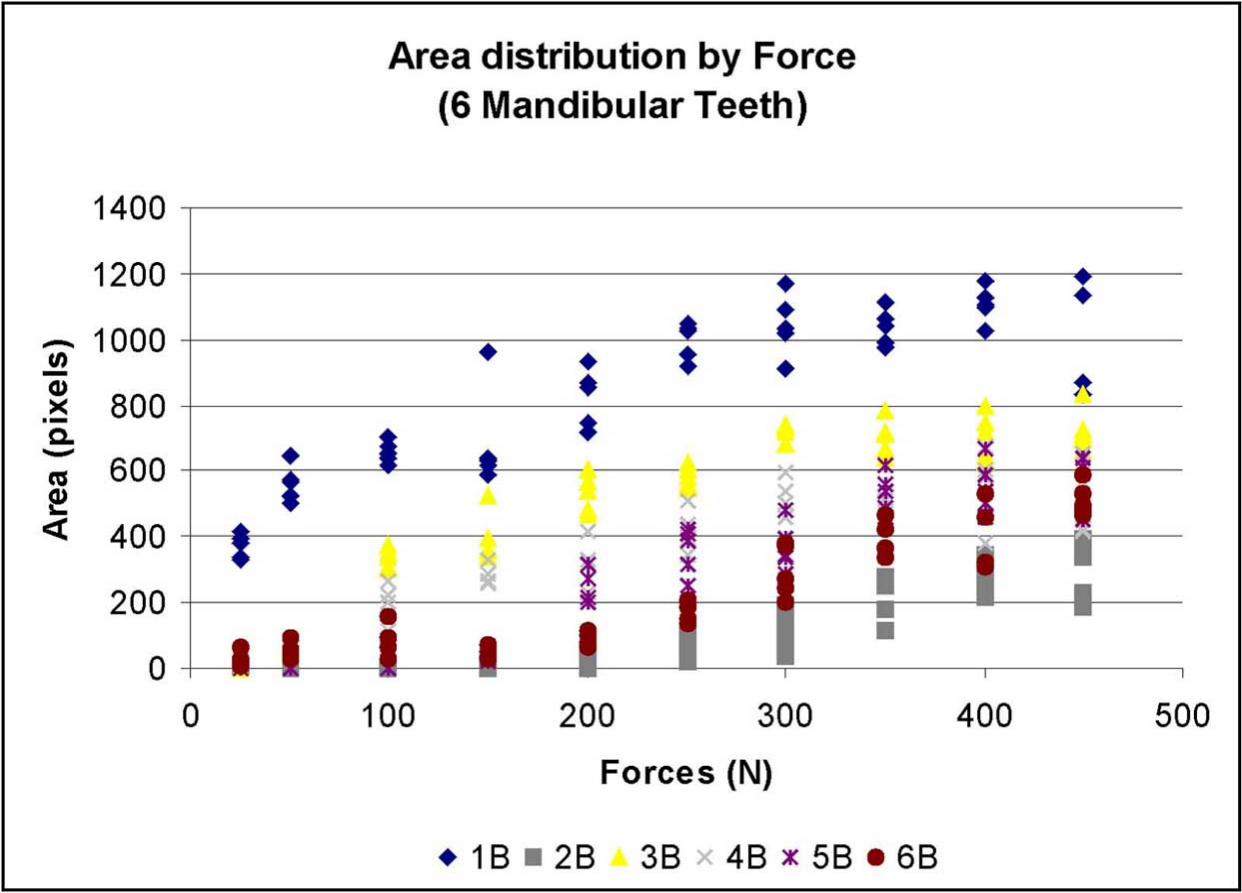
Mandibular teeth: Force vs. Mark Area. – Wide range of mark areas present at each force.

**Fig. (9):**
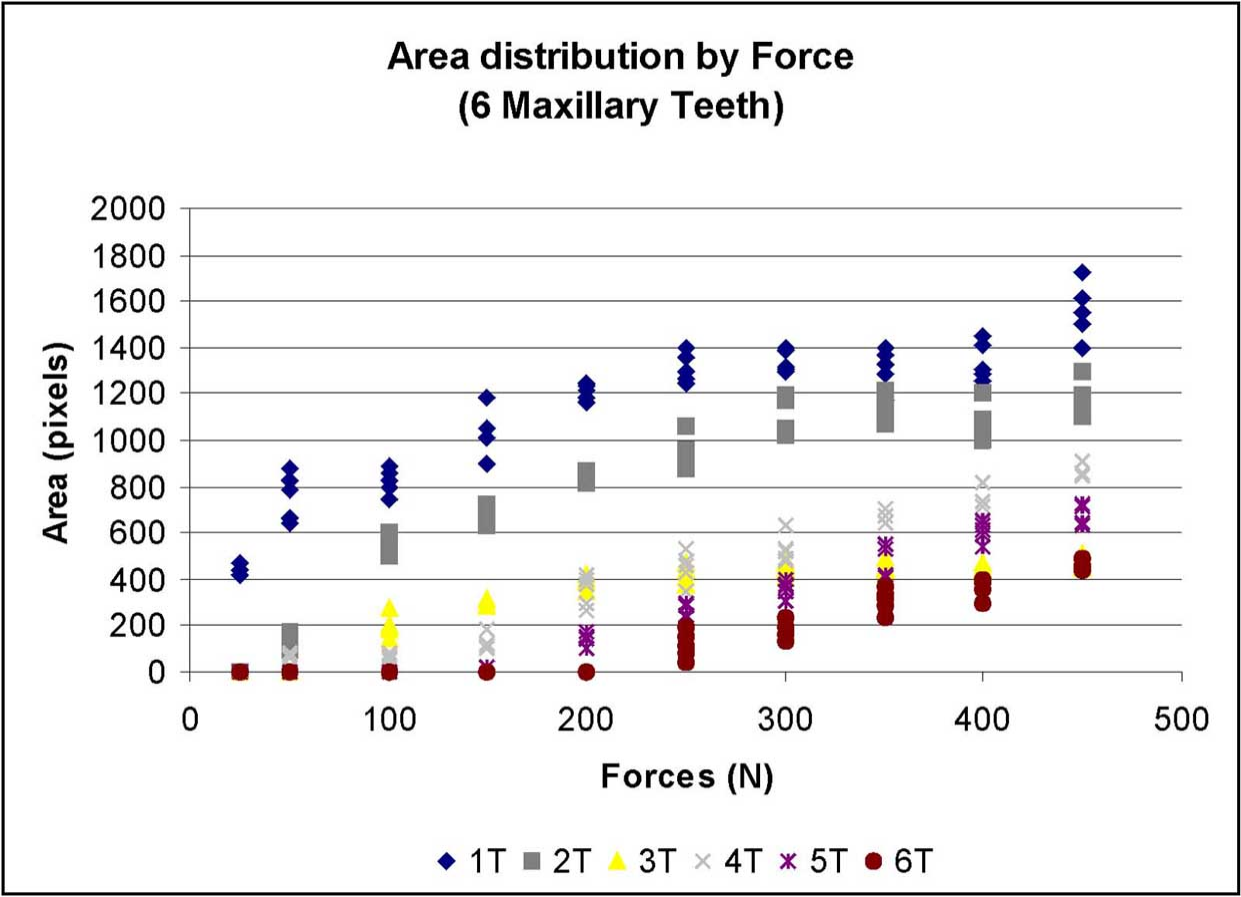
Maxillary teeth: Force *vs*. Mark Area.–A wider range (than mandibular teeth) of mark areas present at each force.

Data was plotted for each of the twelve marks (Figs. **6** and **7**). A best-fit curve and regression analysis was performed. Data was also grouped plotted by each load level to calculate descriptive statistics (Table **1**) and (Figs. **8** and **9**).

Because there was so much variation in the mark area between teeth, the data was grouped together by tooth. To test the null hypothesis of the mark areas on different teeth did not significantly differ, the means and standard deviations were re-calculated (Table **2**). Since all 12 teeth were subjected to exactly the same loads, the Student’s t-test was employed to determine if the mark areas were the same or significantly different at each load (Table **3**).

A review of the plots and best fit curves in Figs. **6** and **7** suggests that the mark area increases in size with increasing load. However, there is very little homogeneity among the mark areas. The individual equations and curves describe very different relationships at each tooth contact; from first order roughly linear relationships (4T, 5B) to third order very non-linear relationships (1T, 1B 2T, 2B, 3T, 3B, 4B, 5T, 6T, 6B). The large standard deviations in Table **1** verify the high variability that is seen graphically in Figs. **6** and **7** where the range of the areas associated with each load is very broad.

Although there is a subtle one-tooth trend towards a positive, non-linear, Correlation between increasing load and increasing Mark area, when more than 1 contact and teeth are observed, this positive non-linear relationship cannot be further demonstrated. This is visualized in the different best fit curves in Figs. **6** and **7**. They are highly variable from contact to contact. Four curves follow a logarithmic-like curve path (2B, 5T, 6T, 6B), 6 curves follow an exponential-like path (1T, 1B, 2T, 3T, 3B, 4B) and two are linear (4T, 5B). It is interesting to note that in some of the high-load regions where the curve is concave down, the mark area is actually deceased with an increasing load (2T, 3T, 3B, 4B). What is most significant is that the 12 curves demonstrate unpredictability of the mark area resultant from linearly increasing loads.

The Student’s *t*-test (Table **3**) illustrated that 14 of 66 comparisons were not significantly different. That is approximately a 21 % agreement, which indicates that about once in five times, equal sized marks actually described equal loads.

During all tests, no gross observable paper failure was found; however, some local indentations or crinkling was observed as paper conformed to the shape of tooth edges.

## DISCUSSION

To measure the variability of dental articulating paper mark area resultant from increasing applied occlusal load, it was necessary to design a test that eliminated from the scientific design the following intraoral variables that can alter articulating paper mark area during an occlusal contact marking procedure: tooth movement, tooth wear under repeated loading conditions, intraoral moisture, angular mandibular movement, and mandibular deformation under loading. Each of these variables would distort the in-vitro behavior of the articulating paper marking mechanics; the methods described meet all these specifications.

It has been advocated that paper marks can be judged and selectively adjusted for load concentration based upon their relative size. Although drawing distinct clinical conclusions from this bench analysis is difficult because the intraoral environment is not exactly replicated, the results of this study do suggest that, when using articulating paper to mark teeth, the operator should not assume the size of the paper markings which can predict the amount of occlusal load. Nor can the operator assume that equal sized markings on nearby teeth represent similar applied occlusal loads.

There is a positive, but usually non-linear, correlation between increasing load and increasing mark area when observing 1 contact only. This implies that if the load to a single occlusal surface was equally distributed across that entire surface, the larger marks would indicate areas of greater load. This may explain, at least in part, why many clinicians and authors advocate that “the bigger the articulating mark, the greater the load.”

An important observation of the data in Figs. **6** and **7** is that the incremental load increase did not result in an equal mark area size increase on any individual contact. Even in the nearly linear best-fit curves (4T, 5B) there is no clear one-to-one relation between load and mark area.

Table **3** shows a 21% agreement between applied load and mark area. This indicates that there is a low probability in a quadrant of marked teeth (or greater numbers of teeth) that similar sized marks will demonstrate equal loads. The results of both the within tooth, and between contact analyses suggest that relative mark area cannot be used reliably to measure relative load.

These findings question the long-standing concepts that mark area predicts the load and similar sized marks demonstrate “evenness and equal intensity” [1,3,5,6,7]. These concepts appear to have been author-advocated premises that have been widely accepted in Dentistry, without any physical evidence to substantiate them as true. To date, there are no published studies in the literature that illustrate articulating paper that has the capacity to measure occlusal load or measure time incrementally. With 600 marks analyzed, the current study reveals that mark area does not reliably describe load, and that similar sized marks do not contain equal load. Although there is more study required to further document the relationship between mark area and applied occlusal load, without this initial mark area analysis, these unsubstantiated author-advocated premises would probably continue to be advocated in dental education.

Extrapolating these in-vitro findings to the clinical procedure of interpreting articulating paper mark area with respect to load applied is difficult because of the many differences between the epoxy cast environment and the intraoral environment. However, the findings in this study do expose, that in a perfect environment, articulating paper mark area is highly unreliable as an indicator of applied occlusal load. Therefore, with all the other variables added into the clinical scenario, it is likely that mark areas variability would likely be higher.

When an operator is preparing to adjust the occlusion, it is customary to mark at least, a quadrant of teeth on both arches simultaneously. This means several teeth in proximity and apposition will bear marks. If the occlusal adjustment objective is to reduce hyper-occlusion and create equal intensity contacts on all teeth [1,2,3,5,6,7] it becomes important to know how the mark areas on different contacts on neighboring teeth compare to each other in relation to the load applied.

The mandible, when tapping teeth together during the action of paper marking, applies a given load to each contacting tooth. The load will vary between taps and between teeth. The marks are imprinted upon the occlusal surface after the series of taps are completed. Then, the operator subjectively (possibly with the aid of shim stock tug) interprets the markings for force content based upon their appearance. It has been taught that the larger marks are subjectively determined to contain more force [1,2,3,5,6,7]. No such conclusion can be made from the current study. Computerized occlusal analysis showed that similar sized and widely distributed marks did not indicate a measurably simultaneous occlusal scheme [8]. It was also shown that, despite their similar size, those same marks exhibited a wide range of forces. Kerstein noted that the smallest marks often demonstrated the highest force and pressure concentrations [9]. The small surface area resulted in poor pressure dissipation, where pressure is the force over the surface area.

This was also confirmed in a sensor force reproduction analysis, where 6 similar sized paper marks, made from occlusal contacts on 2 articulated epoxy casts, demonstrated variable force and pressure content [9]; further evidence that similar sized marks do not contain equal load and/or pressure. These statements are contrary to what has been previously advocated with respect to mark area appearance and the mark’s occlusal load content [1,2,3,5,6,7].

An interesting finding was that 6 of the best-fit curves were “concave down” indicating paper compression at higher loads yielded smaller markings. The 4 “concave up” curves indicate that at lower occlusal loads, paper mark size may not show any appreciable increase in mark area as load increases. Lastly, the 2 near-linear curves indicate that the mark area may increase in size from increasing applied load. This occurrence was the least observed. The near-linear data illustrated that any mark area increase did not equal the load increase. Assuming this same phenomenon occurs clinically some of the time with a variable load range of patient controlled taps, the operator’s choice to adjust larger marks would be correct. However, based upon the study data, this is unlikely to occur clinically as only 21% of the time did similar loads result in similar mark area.

The data illustrates many contact size comparison examples of where similar sized mark areas did not represent similar load. In Fig. **6**, where contacts #s T2 and T3 are similarly sized 400 pixel marks, contact T2 equaled 100 N while neighboring contact T3 equaled 325 N. Here the same sized mark described a 200N load difference between contacts T2 and T3. In Fig. **7**, where contacts numbers T4 and T5 are similarly sized 600 pixel marks, contact T4 equaled 325 N while neighboring contact T5 equaled 400 N. Here, the same sized mark described a 75N load difference between contacts T4 and T5. Lastly, in Fig. **7**, where contacts numbers B4, B5, and B6 are neighboring similarly sized 400 pixel marks, contact B4 equaled 225 N, contact B5 equaled 300N, while contact B6 equaled 400 N. Here the same sized mark described 3 different loads ranging across 175N, which equals 35% of the human occlusal load range. Further inspection of Figs. **6** and **7** will reveal additional examples where equal sized marks do not represent equal load.

In Figs. **8** (mandibular teeth) and **9** (maxillary teeth), which are plots of the Load Applied (x) vs. the Mark Area (y), the mandibular contacts demonstrate a wide range of mark areas for each force applied. At 250 N the range was from near 0 to over 1000 pixels. At several contacts there were negligible marks made until the load reached 200 N, while other contacts produced an area greater than 400 pixels with only 50 N of load applied. The maxillary contacts exhibited an even greater range of mark area per load (from near 0 to 1400 pixels at 250 N of force). There is no consistent relationship seen in either arch. Both of these figures illustrate the large disparity in the mark area between different contacts at the same load. In a clinical situation therefore, it would be quite difficult for an operator to accurately discern differing occlusal loads by comparing contact mark area on neighboring (cusps and) teeth.

### Limitations

Only 1 type of commonly used articulating paper was used in this study so extrapolations of the behavior of other paper/ribbon types cannot be universally made. The results do not necessarily reflect other types, and/or thicknesses of differing commercially available articulating papers. Further study using dental articulators, to simulate the angular movement of the mandible, in combination with other papers of alternate thicknesses and substrates, will be required to determine if the trends observed in this in-vitro study are observed with other types of papers. In an attempt to obtain statistically worthwhile data, the solely employed horseshoe articulating paper was tested to generate 600 paper marks for analysis. In this study, the complexities of the anatomical and physiological aspects of the human teeth which rest in the hydrodynamic environment of the Periodontal Ligament were purposefully not duplicated. A final limitation was when subjectively defining and sketching the boundary of the mark area. However, in pilot testing, it was apparent that ImageJ automatic boundary selection was less accurate than manual boundary selection. Five readings were taken of each boundary to attempt to reduce this error. It was easier to identify the boundaries of the red markings versus the blue markings.

## CONCLUSION

In this bench analysis, a linear relationship between applied load and articulating paper mark area could not be found. This was due to the high degree of mark area variability observed at each test load between differing teeth and contacts. Additionally, similar sized mark areas did not represent similar applied occlusal loads. These findings question the long-standing dental premises, that the size of an articulating paper mark indicates its’ load content, and that similar sized marks indicate similar applied occlusal loads. The results of this study suggest that the size of an articulating paper mark may not be a reliable predictor of the actual load content within the occlusal contact. However, the general trend in this work shows increasing mark area with increasing load.

## Figures and Tables

**Table 1:** Means and Standard Deviations of the Sum of the Corresponding Top and Bottom Mark Area (Pixels) Data Grouped by Load Level (Newtons) - Rounded to the Nearest Pixel

	Force (N)				
	25	50	100	150	200
**Mean**	69	136	243	300	414
**SD**	151	246	281	333	364
	**250**	**300**	**350**	**400**	**450**
**Mean**	505	588	644	732	733
**SD**	378	382	338	367	359

**Table 2:** Means and standard deviations of pixel area data grouped by tooth- rounded to the nearest pixel

	1T	2T	3T	4T	5T	6T
**Mean**	1112	768	311	381	257	142
**SD**	338	407	117	298	257	170
	**1B**	**2B**	**3B**	**4B**	**5B**	**6B**
**Mean**	834	97	475	327	263	208
**SD**	256	124	266	208	234	179

**Table 3:** Student’s t-test comparing mark areas at all twelve teeth. ^* ^indicates p < 0.05, significant; the probability of a difference between the Mark Areas of different teeth is > 95 %. # indicates not significant; the probability of a difference between the Mark Areas of different teeth is ≤ 95 %

	1T	2T	3T	4T	5T	6T	1B	2B	3B	4B	5B
**1T**											
**2T**	*										
**3T**	*	*									
**4T**	*	*	#								
**5T**	*	*	#	*							
**6T**	*	*	*	*	*						
**1B**	*	#	*	*	*	*					
**2B**	*	*	*	*	*	#	*				
**3B**	*	*	*	#	*	*	*	*			
**4B**	*	*	#	#	#	*	*	*	*		
**5B**	*	*	#	*	#	*	*	*	*	#	
**6B**	*	*	*	*	#	#	*	*	*	*	#
